# Skin-to-skin SDF positioning: The key to intersubjective intimacy between mother and very preterm newborn—A pilot matched-pair case-control study

**DOI:** 10.3389/fpsyg.2022.790313

**Published:** 2022-10-11

**Authors:** Aude Buil, Carol Sankey, Laurence Caeymaex, Maya Gratier, Gisèle Apter, Lisa Vitte, Emmanuel Devouche

**Affiliations:** ^1^Laboratoire de Psychopathologie et Processus de Santé (LPPS UR 4057), Université Paris Cité, Paris, France; ^2^NICU Service de réanimation néonatale, Hospital Center Intercommunal De Créteil, Créteil, France; ^3^Faculté de santé - Université Paris Est Créteil, Créteil, France; ^4^Université Paris Nanterre, Nanterre, France; ^5^Service de pédopsychiatrie universitaire, Hospital Group Du Havre, Le Havre, France

**Keywords:** first skin-to-skin contact, SDF positioning, mother-very preterm newborn interaction, innate intersubjectivity, Kangaroo Care, NICU, vocal production, very premature birth

## Abstract

**Background:**

Skin-to-skin contact (SSC) has been widely studied in NICU and several meta-analyses have looked at its benefits, for both the baby and the parent. However, very few studies have investigated SSC’ benefits for communication, in particular in the very-preterm newborn immediately after birth.

**Aims:**

To investigate the immediate benefits of Supported Diagonal Flexion (SDF) positioning during SSC on the quality of mother—very-preterm newborn communication and to examine the coordination of the timing of communicative behaviors, just a few days after birth.

**Subjects and study design:**

Monocentric prospective matched-pair case-control study. Thirty-four mothers and their very preterm infants (27 to 31 + 6 weeks GA, mean age at birth: 30 weeks GA) were assigned to one of the two SSC positioning, either the Vertical Control positioning (*n* = 17) or the SDF Intervention positioning (*n* = 17). Mother and newborn were filmed during the first 5 min of their first SSC.

**Outcome measures:**

Infants’ states of consciousness according to the Assessment of Preterm Infants’ Behavior scale (APIB). Onset and duration of newborns’ and mothers’ vocalizations and their temporal proximity within a 1-s time-window.

**Results:**

In comparison with the Vertical group, very preterm newborns in the SDF Intervention Group spent less time in a drowsy state and more in deep sleep. At 3.5 days of life, newborns’ vocal production in SSC did not differ significantly between the two groups. Mothers offered a denser vocal envelope in the SDF group than in the Vertical group and their vocalizations were on average significantly longer. Moreover, in a one-second time-frame, temporal proximity of mother-very preterm newborn behaviors was greater in the SDF Intervention Group.

**Conclusion:**

Although conducted on a limited number of dyads, our study shows that SDF positioning fosters mother-very preterm newborn intimate encounter during the very first skin to skin contact after delivery. Our pioneer data sheds light on the way a mother and her very preterm vocally meet, and constitutes a pilot step in the exploration of innate intersubjectivity in the context of very preterm birth.

## Introduction

In the 1970s, scientists brought evidence that, before they actually begin to speak, newborns, as well as young infants, experience intersubjective awareness. Their thoughts develop through the sympathetic and timely engagement with the expressive behaviors of a sensitive parent (Bruner, 1977; Trevarthen, 1977; Bateson, 1979; Brazelton, 1979; Brazelton et al., 1979). The centrality of interpersonal synchrony was highlighted through pioneer studies using videotape recordings of mother–infant interactions (Trevarthen, 1977; Tronick et al., 1977; Feldman et al., 1999; Jaffe et al., 2001). The present study falls within the psychobiological theory of intersubjectivity (Trevarthen, 2012, 2016). The theory of innate intersubjectivity claims that “a child is born with motives to find and use the motives of other persons in ‘conversational’ negotiation of purposes, emotions, experiences, and meaning. The efficiency of sympathetic engagement between persons signals the ability of each to ‘model’ or ‘mirror’ the motivations and purposes of companions, immediately” [Trevarthen, 1998 (in Braten) p. 16]. In this perspective, achieving a conversation at birth rests on an efficient mobilization of the neonate’s internal resources (self-regulation), as well as on the efficient sympathetic regulation of a partner. The theory of innate intersubjectivity considers newborn and partner as an intrinsic unit, the dyad. It is especially activated during intimate dyadic free play. In such face-to-face, eye-to-eye intersubjective emotional communications, infant and mother intently look at and listen to each other. Thereby, they synchronize and regulate each other’s emotional states. During these protoconversations, the partners’ emotions are expressed and actively perceived in spontaneous, reciprocal, and rhythmic-turn-taking interactions (Trevarthen, 1993). In this perspective, the dyad becomes the place where they meet, as well as where they miss each other. Every time they meet or miss each other results from an intelligent combination of their ability to perceive each other’s motives. In her tribute to Stern, Beebe (2017, p. 234) stated that “the meaning of the behavior is co-created,” recalling that the way communication is bidirectionally regulated is specific to a particular dyad. Feldman et al. (2011) developed a similar neurophysiological model, including physiological synchrony from birth: when mother and child are physically close, they synchronize their breathing and heart rate. This provides a basis for later “social synchrony,” in which partners meet and miss one another and respond during shared interactions. But sometimes birth comes earlier than expected and requires hospitalization, thereby separating mother and child and medicalizing their first relations. The present study investigates the essential question of how and to what extent the quality of communication can be supported from the start in the context of very preterm birth.

### Before term birth

For the fetus, pregnancy is a time for chemosensory learning, which helps him adapt to his new after-birth environment (Lickliter, 2000). The uterine environment provides plentiful multisensory stimulations. The fetus can experience vestibular, somaesthetic, auditory, chemical and olfactory stimulations, simultaneously or sequentially (Pinéda et al., 2017a).

*In utero*, the fetus moves intentionally, with controlled timing and sequencing, anticipating sensory confirmation of self-related effects (Piontelli, 2010). The fetus is bathed in an environment of vestibular, somatosensory, tactile, and auditory rhythmic stimuli, through the mother’s breathing, heartbeats, walking, dancing, running, speaking, singing, etc. (Provasi et al., 2021). After 20 weeks, some fetuses make self-touching gestures with their left hand, which may signal sympathetic emotional attitudes with a mother’s feelings of stress, and respond positively to her voice (Reissland and Kisilevsky, 2015). Various sounds are present in the uterus, with frequencies and intensities that are low yet detectable by the fetus (Philbin, 2017). When the mother speaks or sings, sound travels through the inside but also through the air, enabling the fetus to perceive her voice from both internal and external sources (Pino, 2016). Fetuses are able to clearly identify the mother’s voice, and could shape mouth movements when they hear someone speaking (Marx and Nagy, 2015). Toward the end of pregnancy, fetuses are particularly attuned to maternal acoustic cues (Ferrari et al., 2016) and they are able to detect, recognize, respond, and remember some characteristics of her voice (Kisilevsky et al., 2003; Voegtline et al., 2013).

### Term birth

Babies are equipped to perceive the world they are born into. Newborns have a holistic perception of others: they perceive faces, voices, body movements, tactile stimuli, smells, and shapes. Evidence suggests that the mother’s voice is one of the major drivers of the intricate intersensory and intermodal connections that are formed and retained in memory in the days following birth (Gratier and Devouche, 2017). The mother’s voice is a salient, consistent and frequent stimulus that provides before-after birth continuity, regardless of the drastic changes linked to the exit of the uterine environment (Lickliter, 2000). From that perspective, the full-term newborn experiences a normal biological continuum between intra- and extra-uterine lives. At birth, the newborn is exposed to multimodal stimulations that form intermodal redundancies (joint and repeated apprehension of the environment with different sensory modalities; Provasi et al., 2014). This gives meaning to the newborn’s environment, making his/her ecological niche. Significant biological stimulations originating from the mother, notably on the olfactory (Varendi et al., 1997) and auditory levels (Doheny et al., 2012), lead to an improvement of physiological and behavioral stability and better adapted responses.

A parent, or any caring elder, can pick up on the infant’s skills to regulate him- or herself as well as the partner, thus enabling the development of a new and unique relationship of intimate familiarity and responsiveness (Brazelton et al., 1979; Trevarthen, 2011; Devouche and Gratier, 2019). In this context, Dominguez et al. (2016) and Boiteau et al. (2021) highlighted a tight coordination in the timing of vocalization between 2 to 4-day-old neonates and their parent. In both studies, neonates demonstrated control over the timing of vocalizations produced with an attentive and affectionate partner, with a 1-s temporal window explaining most baby-parent vocal contingencies. However, to date, no study has explored temporal coordination between the newborn and a partner in the context of preterm birth.

### Preterm birth

Preterms have been denied part of their prenatal chemosensory learning time, and deprived of the typical biological continuum between pre- and postnatal life. In addition, after birth, they have to face the incubator’s harsh environment that combines sensory deprivations, over-stimulations and inappropriate stimulations (Lickliter, 2000), which do not match their pre-organized sensory expectations. Preterm babies receive many sensory stimulations that are not adapted to their sensory maturation level, including more and higher levels of auditory stimulations (machine noises and rings, voices that are transformed and amplified by the incubator’s walls) and reduced vestibular stimulation, at a time when they should be benefiting from filtered auditory stimulations, and regular vestibular stimulations provided by the mother’s movements (Lickliter, 2000). They are also likely to be subjected to visual over-stimulations (bright lights) sometimes necessary in highly technical care. Furthermore, they are confronted to many unpleasant (antiseptic agents) but very few familiar or maternal smells (Marlier and Schaal, 2005). The preterm newborn is thus exposed to early sensory experiences that are atypical, both in quality and quantity, and that occur during the most critical period for the nervous system’s development (Pinéda et al., 2017b; Montagna and Nosarti, 2016).

At birth, preterms’ motor and interactional skills are fragile, which makes it is more difficult for them than for healthy term babies, to initiate an exchange and respond actively (Muller-Nix et al., 2004). However, they can discriminate between two stimuli, associate events, and become habituated to external stimulations. The preterm infant does receive environmental input and plays an active part in the interactive system (Provasi, 2019). Nevertheless, interactions between mothers and preterm children often involve poorer maternal adaptation to the infant’s signals, leading to less maternal touch, as well as fewer vocalizations and gazes (Feldman, 2006; Forcada-Guex et al., 2011).

Preterm birth is associated with high stress and anxiety levels in parents (Segre et al., 2014), including post-traumatic stress (Forcada-Guex et al., 2011; Anderson and Cacola, 2017). A correlation has been evidenced between maternal and paternal stress, as well as between parental stress and the quality of father–mother–infant interactions in premature (Gatta et al., 2017). In the meta-analysis of Grote et al. (2010), 39% of mothers with a preterm baby presented postpartum depressive (PPD) symptoms. De Paula Eduardo et al.’s (2019) systematic review analyses the studies that explored preterm birth as a risk factor for postpartum depression in the last 10 years, and provides evidence of higher risk for PPD among mothers of preterm infants up to 24 weeks after childbirth. Stressful or traumatizing events have been shown to slow down oxytocin release, which inhibits the mother’s empathetic abilities (Feldman, 2015). Indeed, mothers of preterm infants provide a less responsive and stimulating environment than mothers of full-term infants (Muller-Nix et al., 2004). Preterm birth could permanently disturb the interactional sphere (Zelkowitz et al., 2009). The analysis of preterms’ neurological and behavioral disturbances highlights the entanglement between biological vulnerability (brain alteration) and environmental factors, such as stress, perinatal pain, and break of the emotional connexion with the mother (Montagna and Nosarti, 2016).

Pierrat et al. (2021, the EPIPAGE 2 study) showed that at age 5, behavioral disturbances were parents’ most frequent preoccupation in France. This study also showed that the level of prematurity is crucial to neurodevelopment: moderate or severe developmental difficulties observed in 27% of extreme preterm children, 19% of very preterm children, and 12% of moderately preterm children. Irrespective of the degree of prematurity, 35% of preterms require adapted care to prevent difficulties from impacting the child’s daily life and learning. More precisely: 50% of extreme preterms, 1/3 of very preterm children and ¼ of moderately preterm children received support in their development (speech therapy, psychomotor education, psychological support …). In the OLIMPE study, an ancillary study to EPIPAGE 2, Cambonie et al. (2017) recorded disorganized interactive behaviors on discharge from the maternity ward (50% for mothers, 33% for preterm babies), which persisted at 6 months (32% for mothers and 26% for preterms).

### Skin-to-skin contact

Skin-to-skin contact is a consistent and reciprocal interaction, which is entirely dedicated to the parent-infant relationship. It is performed routinely around the world, starting from birth, as part of intensive care in neonatal units (Nyqvist et al., 2010). Skin-to-skin contact is offered during hospitalization, as part of family-centered care programs, as a support of the subjective experience of parenthood (Roué et al., 2017). This natural relational opportunity helps parents develop a sense of responsibility that is often on hold while the infant is in medical care. Skin-to-skin contact, along with infant-directed singing, allows mother and child to synchronize rhythmically and provides an envelope that enables the tuning of different rhythmic stimulations (Markova et al., 2019). Supporting mutual synchrony through SCC is in line with Thomson et al.’s (2013) recommendation to include some sense of coherence into the neonatal environment, by giving parents a central role in the decision-making process and supporting their engagement to care for their child.

In the specific context of a preterm birth, SSC has immediate physiological and neurobehavioral benefits (Feldman and Eidelman, 2003): it facilitates the vulnerable newborn’s adaptation to extra-uterine life (Kristoffersen et al., 2016). Longer-term benefits include better psychomotor (Feldman et al., 2002) and cognitive development (Charpak et al., 2005; Feldman et al., 2014). Mothers and fathers of preterm newborns experience an increase of oxytocin release and a reduction in cortisol and stress responses during SSC, as well as facilitated breastfeeding (Cong et al., 2015; Mörelius et al., 2015). Mothers’ psychological benefits include reduced stress and postpartum depression risk (Athanasopoulou and Fox, 2014; Zhao and Zhang, 2020). Additional benefits have been evidenced on parents’ affective and interactive behavior at corrected term, as well as on the quality of their attachment (Tallandini and Scalembra, 2006; Moore et al., 2016; Feldman et al., 2014). Furthermore, SSC may promote parental presence, even in poor reception conditions (Raiskila et al., 2017).

Indeed, SSC has been widely studied (Charpak et al., 2005) and several meta-analyses or meta-syntheses have investigated its implementation (Seidman et al., 2015; Chan et al., 2016) or its benefits (Moore et al., 2016; Lawn et al., 2010; Mori et al., 2010; Anderzen-Carlsson et al., 2014; Johnston et al., 2017; Conde-Agudelo et al., 2011).

There has been a real paradigm shift toward family-centered care (Franck and O'Brien, 2019; Skene et al., 2019). In their paper presenting eight principles for patient-centered and family-centered care for newborns in the Neonatal Intensive Care Unit (NICU), Roué et al. (2017) listed the main benefits of SSC, however omitting to mention potential benefits for communication. Studies investigating this aspect are scarce. In their systematic review on the effects of early communication intervention on the speech and communication skills of preterm infants in the NICU, Harding et al. (2019) identified five studies, all conducted on very or extremely preterm babies, with outcome measures collected 1 month after birth (one study) and at 3 months CA or more (four other studies). Importantly, four of these studies correlated infant and mother measures but none actually focused on communication during SSC. Only Caskey et al. (2014) investigated the timing and coordination of parent-preemie communications, through a turn-taking measure computed by the LENA system, with a 5-s time window of temporal contingency. Authors considered any block that contains both infant and adult speech as conversational and found that infants responded preferentially and more frequently to their mothers compared with their fathers. However, their measure of coordination timing remained less precise than the one used by Dominguez et al. (2016) and Boiteau et al. (2021).

The search terms used for the Harding et al. (2019) review excluded several studies, such as Tallandini and Scalembra (2006), Feldman et al. (2014), or Buil et al. (2016) that nonetheless focused on mother-preemie communication. The study conducted by Buil et al. (2020) precisely aimed to investigate how communication could be improved during SSC, by modifying SSC positioning. Considering that mothers often complained about the impossibility to look at their baby’s face, because of the vertical positioning of their child on their chest (leading them to sometimes prefer an arm-holding cuddle), authors investigated how an innovative kangaroo Supported Diagonal Flexion (SDF) positioning influenced mother and infant well-being and communication during SSC, while maintaining a safe and preventive preterm posture (Buil et al., 2016). This positioning was developed in a high-tech NICU according to recent experts’ recommendations for promoting a “semi reclined positioning” (Ludington-Hoe et al., 2008; Nyqvist et al., 2010). SDF positioning was also found to reduce the risk of postnatal maternal depression and to promote and prolong SSC sessions (Buil et al., 2019). Moreover, Buil et al. (2020) tested the kangaroo SDF positioning 18 days after a very preterm birth and showed that SDF positioning improves the mother’s ability to perceive her infant’s behaviors such as vocalizations, smiles or eyes openings, as well as to respond in a timely manner. Since its creation, other teams have investigated potential benefits of skin to skin in SDF position, either looking at “the influence of a skin-to-skin lullaby on the stability of maternal behavior and on the tonic emotional manifestations of the preterm infant” (Provasi et al., 2021) or at the “vocal responsiveness of preterm infants to maternal infant-directed speaking and singing during skin-to-skin contact (Kangaroo Care) in the NICU” (Carvalho et al., 2019). However, in these studies, observations were not made immediately after birth. Therefore, the essential question of how and to what extent SDF positioning enhances the quality of communication from the start is yet to be examined. In this direction, our team has previously explored touch and maternal vocal behavior during the first skin to skin in a sample of our population. In this position, mothers display more active, securing and affectionate touch, favoring a quality early reunion, free from over-stimulations, as reflected by drowsiness and less agitation in the baby (Buil et al., 2017a). Moreover, from the very first minutes of skin to skin, mothers in SDF provide a denser and more musical sound envelope (Buil et al., 2017b).

Given our current knowledge on the effects of temporal coordination during communication on infant development, particularly in the neonatal period and infancy, the question of mother-preterm communication is decisive. The first minutes of the very first skin to skin are the physical and emotional reunions of mother and child after birth in a potential traumatic context. Nonetheless, current literature regarding the first skin to skin in the NICU focuses mainly on its secure feasibility (Linnér et al., 2020), the moment of and impact on its implementation during hospitalization (Mörelius et al., 2012; Blomqvist et al., 2013), its physiological benefits for the preterm (Cadwell et al., 2018; Gere et al., 2021; Pandya et al., 2021) or parents’ feelings (Maastrup et al., 2018). The data collected in this pioneering study shed light on what actually happens between mothers and their babies born prematurely during their first skin to skin.

The present study aimed to better characterize the vocal meeting of the mother and her very preterm newborn, during the first minutes of their first ever skin to skin. We also further examined the immediate benefits of SDF positioning during the first SSC on the quality of mother—very-preterm infant communication. We hypothesized that, compared to Vertical positioning and as early as 3–4 days after birth, SDF positioning would, by increasing opportunities for eye contact, improve the mothers’ ability to recognize her infant’s signals, thus enabling a more timely feedback. Finally, we aimed to examine how the communicative behaviors of mothers and very-preterm infants were coordinated in time during SSC, just a few days after birth.

## Materials and methods

### Participants

The study was conducted between May 2015 and June 2016, in a level III NICU at the *Centre Hospitalier Intercommunal de Créteil* (France). Among the 90 very preterm babies (27 to 31 + 6 weeks’ gestation) admitted during the inclusion period, 53 met parent and child’s inclusion criteria [living in the geographical area considering the longitudinal follow-up, no multiple birth > 2, no neurological complication due to several vascular hemorrhage (IVH grade III, or IV)], no severe medical conditions, no incapacitated physical or psychological illness in the mother. Seven mothers refused to participate, and two inclusions were missed. Among the remaining 44 births, two were lost during follow-up and eight were multiple births which were not included in the present report. The final sample included 34 very preterm infants and their mothers. The first 17 dyads were offered SSC positioning, as usually practiced in the participating NICU (Vertical Control Group). The following 17 dyads were offered the Supported Diagonal Flexion (SDF) Intervention positioning and these were matched to the first 17 dyads on newborns’ gestational age at birth and weight at birth (see Buil et al., 2020 for the detailed method). Participants’ socio-demographic, Ob/Gyn, delivery and birth data were obtained from medical files.

The present study is part of a longitudinal follow-up from very preterm birth until 3 months corrected age. The data presented constituted the first data collection point of this prospective monocentric matched-pair case-control study. Some data from our study of mother-very preterm communication at 18 days post-partum have already been published in a previous paper (Buil et al., 2020). Measures of the mothers’ risk of depression (made before the first SSC session), were comparable for the SDF positioning group and the Vertical positioning group, with EPDS mean scores of 13.8 and 12.9, respectively (Buil et al., 2019).

### Skin-to-skin positioning in each group

**Figure 1:**
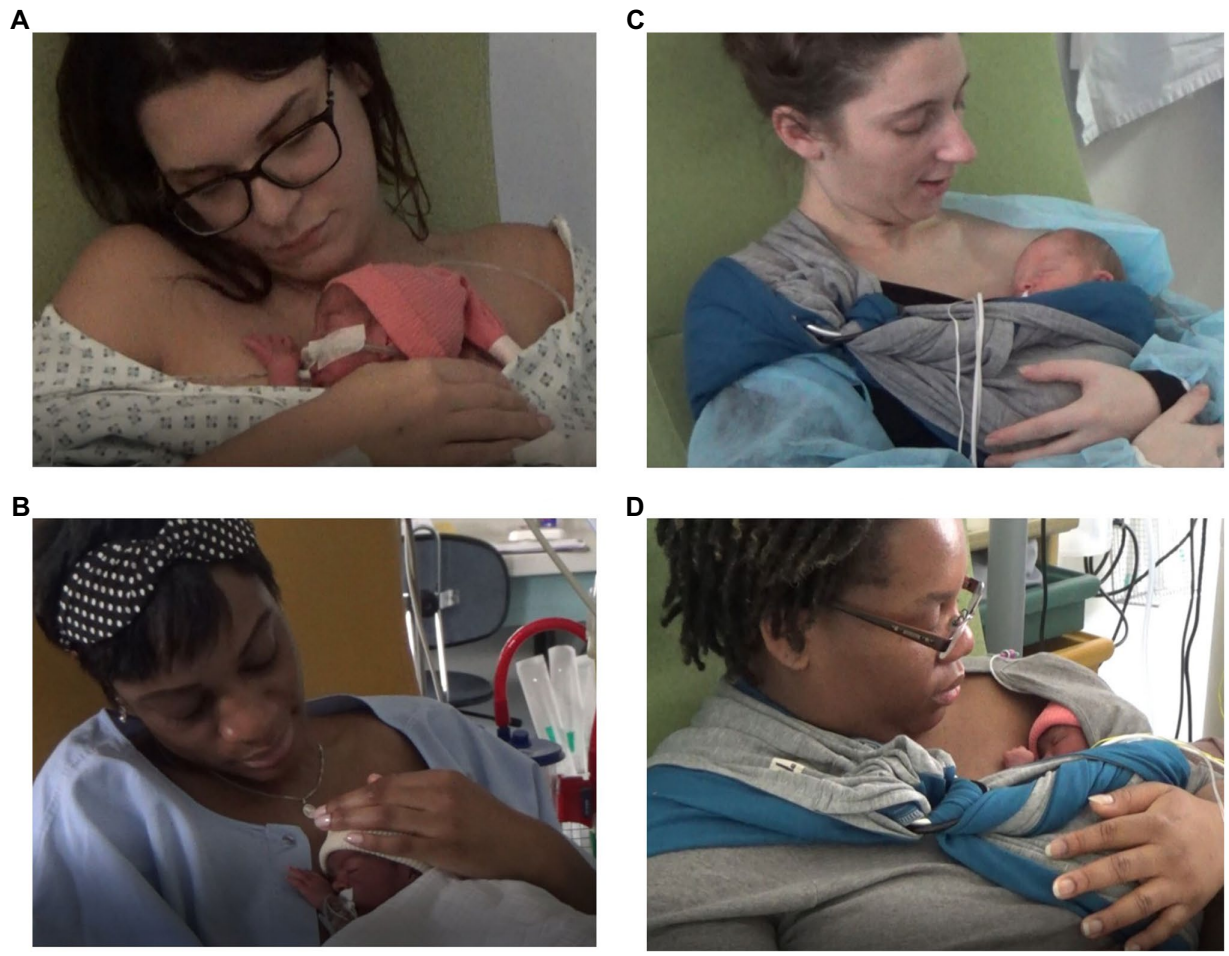
Vertical control positioning **(A,B)** and Supported Diagonal Flexion (SDF) intervention positioning **(C,D)** during the very first SSC after very preterm birth.

In the Vertical Control Group, preemies were positioned chest to chest between the mother’s breasts, at the center and on the median line of the mother’s torso, in an upright position, with a breastfeeding nursing pillow (see Figures 1A,B). According to Kangaroo Mother Care guidelines, the head is turned to one side and in a slightly extended position which keeps the airway open. Moreover, the hips should be flexed and extended in a “frog” position; the arms should also be flexed (World Health Organization, 2003).

In the SDF Intervention positioning (see Figures 1C,D), the baby is naked, off-center and semi-reclined on the mother’s chest, chest to chest (Buil et al., 2016). In this intermittent kangaroo mother care, the choice of on which side of the body (right or left) the baby’s head is positioned is up to the mother. The SDF position is characterized by a slightly flexed body axis, with the limbs retracted in a preventive posture and the head in line with the body axis to prevent side-to-side toppling and to free respiratory permeability (Nyqvist et al., 2010), moderately externally rotated hips in flexion-abduction (Vaivre-Douret et al., 2004), with adducted shoulders (Ferrari et al., 2007). The baby’s head is located between the mother’s nipple and clavicle and oriented toward the mother’s face. His/her arms and legs are flexed, in a naturally adopted asymmetrical tonic neck posture (Casaer, 1979), according to the baby’s gestational age and comfort. The baby is positioned on the mother’s naked chest, a baby wrap adjusted around the two is used to support and help maintain the baby’s posture and to relieve the mother. For the study purpose, we chose The Little-Wrap-Without-A-Knot is a knitted fabric, specially made for Baby Wearing. On one end of the cloth are sewn two metal rings. The Fabric has a special density of 300 gr/m^2^. It is made of a two-faced Viscose certified Oeko-Tex 100. The special mechanism of this knitted fabric, allows it to slightly stretch, and to ensure a soft touch that respects the preterm baby’s skin. The Little-Wrap-Without-A-Knot is put as a loop around the mother’s body. It goes from shoulder to opposite hip and back up to the shoulder, in an asymmetric diagonal. The end of the fabric passes through the rings that creates a buckle effect. This adjustment system ensures a precise fit between the two morphologies and to maintain the desired position of the baby.

In both positionings, the mother was comfortably seated in an adjustable armchair, her back inclined 40°, with a toe-clip, dim light and a quiet atmosphere; she had the choice to wear clothes or not. In SDF Intervention positioning, the baby wrap was placed over the mother’s clothes or on her naked chest.

### Apparatus

Mother and neonate were filmed during their very first SSC, on average 3.7 days after very preterm birth, i.e., on average 29.4 weeks GA (see Table 1). For each dyad, vocal interaction was analyzed only during the first 5 min. Indeed, mothers were particularly active during the first minutes of SSC care, before giving way to mutual relaxation. Thus, 5 min recordings allowed sufficient data collection, but left plenty of intimacy time to the dyad. Mother-neonate dyads were filmed using a single camcorder mounted on a tripod and sound recordings were obtained using a shotgun microphone. Video and audio recordings were synchronized. The camcorder was placed in front of the dyad, in order to frame the mother’s chest and the baby’s entire body, as well as their faces.

**Table 1:** Infant’s’ characteristics at birth and at first skin to skin, according to type of SSC positioning.

	**SDF intervention group**		**Vertical control group**	
	*N* = 12	*N* = 11
	Mean (*SD*)	*N* (%)	Mean (*SD*)	*N* (%)
**Birth data**				
Gender				
Girl		8 (67%)		7 (64%)
Weight (g)	1,079 (266)		1,126 (306)	
<1,000		5 (42%)		4 (36%)
1,000–2000		7 (58%)		7 (64%)
Gestational age(w)	29.4 (2.7)		29.4 (2.4)	
<28		3 (25%)		2 (18%)
28 to <32		9 (75%)		9 (82%)
Spontaneous breathing				
Yes		3 (25%)		3 (27%)
**Data at first skin to skin**				
Age (days)	3.8 (2.0)		3.7 (2.1)	
Weight (g)	994 (203)		1,048 (290)	
<1,000		6 (50%)		4 (36%)
1,000–2000		6 (50%)		7 (64%)

### Video and acoustic analyses

Video analysis was performed using The Observer XT with a precision of 40 ms (25 images per second). Vocal data were analyzed with the software Audacity.

### Coding of newborns’ states of alertness

Newborns’ state of alertness was coded with frame-by-frame video microanalysis using the software The Observer XT^1^ and following APIB’s state configurations (Als et al., 1982): (1) deep sleep state, (2) active sleep state, (3) drowsiness state, (4AL) low awake state, (4AH) high awake state, (4B) quiet awake state, (5) active awake state, and (6) crying state. We included an additional “undefined” category, when the infant’s state of alertness could not be coded because the newborn’s face and body could not be viewed in the video. Coding was carried out by a perinatal professional with NIDCAP certification (Newborn Individualized Developmental Care and Assessment Program). We recorded the time of onset and duration of each of these states of alertness.

### Coding of newborn and maternal vocalizations

Maternal and newborn vocalizations were coded using the software Audacity.^2^ Based on the visualization of sonograms and audio guidance, a segmentation into two types of events was made: maternal vocalization and newborn vocalization, (Gratier et al., 2015; Dominguez et al., 2016), both defined as the production of vocal sound that was either continuous or included unvoiced segments of <300 ms. If a pause following an audible vocal sound was >300 ms, two successive vocalizations were coded. Vegetative sounds produced by infants such as burps, growls or hiccups, noise from the environment and vegetative sounds produced by mothers, such as coughs, were not coded. Our criterion was more flexible than that used with full term babies, in order to include more of the preemies’ voiced demonstrations.

### Coding of turn-taking sequences

A turn-taking sequence was defined as a sequence of vocalizations involving at least one alternation between interactive partners, which is expressed as follows. When more than one alternation occurred, with intervening pauses between the two partners, the number of pauses coincided with the number of turns in the sequence (e.g., two turns: “newborn vocalisation-pause-mother vocalisation-pause-newborn vocalisation”; three turns: “newborn vocalisation-pause-mother vocalisation-pause-newborn vocalization-pause-mother vocalisation” and so on). A turn-taking sequence ended when the same partner produced at least two successive vocalizations, or when the pause following a vocalization exceeded 3,000 ms in accordance with previous studies (Stern, 1985; Gratier et al., 2015; Dominguez et al., 2016; Boiteau et al., 2021).

### Training and reliability

A single coder was responsible for coding states of consciousness with the Observer software and two coders for coding the vocal exchange with the Audacity software. None of the coders were aware of the aim and hypotheses under investigation. For this reason, training was performed on randomly chosen dyads both from the SDF and Vertical positioning groups. The training of coders consisted in three steps. First, coders were trained to use the coding template on 4 dyads under the supervision of the researchers. During the second step, each trainee was invited to code 4 additional dyads alone. At the end of this step, the coding was checked and discussed with the supervisor. The third step consisted in reapplying the second step.

Twenty five percent of the data set, chosen randomly, was double-coded. Inter-coder reliability (Pearson product–moment correlations) regarding the number of behaviors ranged from 0.82 to 0.92 depending on the behavior. Onset positions were considered identical if they occurred within 80 ms (i.e., two frames); thus, measures of behavior duration had an error tolerance of up to 160 ms. Both coders correctly identified 77.2% of all onset positions within the subset of double-coded sequences.

### Ethical considerations

All mothers were offered to participate to the research study on a voluntary basis, within the first 2 days postpartum, and in all cases before the first SSC session. Every mother was informed of the research by a letter in the NICU. Mothers gave a written informed consent before participating. Written informed consent was obtained from the mothers for the publication of any potentially identifiable images or data included in this article. An initial information-based meeting was organized prior to data collection. This research was approved by the French Local Ethics Consulting Committee for the Protection of Persons (IRB n°2015120001072).

### Statistics

All analyses were performed using Stata for Windows (version 14; StataCorp). Sociodemographic data, previous obstetrical and delivery data and infants’ characteristics at birth and at first skin to skin contact were compared in both groups, with either a *t*-test or a chi-square test, depending on the measure. The number of vocalizations and their duration were analyzed through a general linear model with an adjustment on age and weight at birth. Parametric tests were not chosen considering these measures did not differ significantly from normality.

## Results

For 11 dyads, either the baby or the mother did not vocalize during the recorded session of SSC. Analysis of the vocal exchange was therefore conducted on 23 dyads, 11 dyads in the Vertical control group and 12 in the SDF Intervention group. Table 2 presents sociodemographic characteristics, obstetrical and delivery data, and Table 1 infant’s data at birth, at first skin to skin, according to SSC positioning. No significant difference was found between the two groups.

**Table 2:** Sociodemographic, previous obstetrical, and delivery data according to SSC positioning.

	SDF intervention group		Vertical control group	
	*N* = 12	*N* = 11
	Mean (*SD*)	*N* (%)	Mean (*SD*)	*N* (%)
**Socio-demographic data**				
Mothers’ age (years)	30.9 (6.4)		30.2 (4.8)	
≥30		9 (75%)		8 (73%)
Living with partner				
Yes		10 (83%)		11 (100%)
Employment status				
Employed		9 (75%)		10 (91%)
**Previous obstetrical history**				
Gestity before current pregnancy	2.3 (1.0)		2.1 (1.0)	
Yes		9 (75%)		7 (64%)
Parity before current pregnancy	0.47 (0.62)		0.88 (0.99)	
Nulliparous		6 (50%)		6 (55%)
Early pregnancy loss	0.88 (0.93)		0.82 (1.24)	
Yes		5 (42%)		3 (27%)
Late pregnancy loss				
Yes		0 (0%)		1 (9%)
Previous history of preterm birth/LBW				
Yes		1 (12%)		2 (18%)
**Current pregnancy**				
Hospitalization during pregnancy				
Yes		5 (42%)		11 (100%)
High risk pregnancy				
Yes		5 (42%)		8 (73%)
Intra uterine growth restriction		3 (25%)		3 (27%)
Hypertension/pre-eclampsia		3 (25%)		5 (45%)
Preterm delivery threat		1 (8%)		4 (36%)
**Current delivery**				
Spontaneous delivery		0 (0%)		0 (0%)
Labor induction		12 (100%)		11 (100%)
Fetal heart rate abnormalities		8 (67%)		4 (36%)
Intra uterine growth restriction		5 (42%)		3 (27%)
Hypertension/Pre-eclampsia		4 (33%)		1 (9%)
Delivery type				
Vaginal delivery		1 (8%)		4 (36%)
Caesarean section		11 (92%)		7 (64%)

### State of consciousness

Infants were mainly in a state of drowsiness (state 3), even more so in the Vertical group (58%) than in the SDF group (35% of time). Active sleep state (state 2) represented around 35% of the time in both groups. However, infants in the SDF group also spent 19% of the time in deep sleep (state 1), while those in the Vertical group spent only 3%. In SDF group, infants spent 58% of the time sleeping (states 1 and 2) compared to 36% in the Vertical group. Distributions were significantly different [*Chi-square* (5) = 116, *p* < 0.0001].

### Newborns’ vocal production

We collected 167 vocalizations: 84 in the SDF group (i.e., on average 7.0 per newborn and 1.4 per minute) and 83 in the Vertical group (i.e., on average 7.5 per newborn and 1.5 per minute, adj *p* = 0.90). Vocalizations’ duration was on average longer in the Vertical group (Vertical: 817 ms, SDF: 537 ms), but not significantly after adjustment on birth age and weight (*adj p* = 0.78). Consequently, newborns’ vocalizations occupied less dialogue space in the SDF group than in the Vertical group (1.3% vs. 1.9% of the 5 min analyzed).

### Mothers’ vocal production

Overall, mothers vocalized twice more in the SDF group (604, i.e., on average 45.3 per mother and 9.1 per minute) than in the Vertical group (269, i.e., on average 24.5 per mother and 4.9 per minute), but the difference was not significant (*adj p* = 0.068). Their vocalizations were on average significantly longer (SDF: 1166 ms, Vertical: 1068 ms, *adj p* = 0.002). Hence, Mothers’ vocalizations occupied more dialogue space in the SDF group than in the Vertical group (20.1% vs. 8.0% of the 5 min analyzed, *p* = 0.007).

### Temporal proximity of mother and newborn vocalizations

Based on a 3-s criterion, 3 preterm newborn vocalizations out of 4 (74%) were at a temporal proximity from a maternal vocalization in the SDF intervention group against 1 out of 2 (47%) in the Vertical control group (*OR* = 3.2, *p* < 0.0001). The difference was still significant when using a 1-s criterion (57% vs. 39%, *OR* = 2.1, *p* = 0.017).

### Turn-taking coordination

Based on a 3-s criterion, 73 mother-newborn turn-taking sequences (TTS) were identified: 44 in the SDF group and 29 in the Vertical group. In all, 75 newborn vocalizations were involved in these sequences, i.e., 45% of the total number of vocalizations collected. Almost all sequences were either one turn “baby–mother” or two turns “mother–baby–mother.” One third of these were two-turns sequences, sensitively but not significantly more in the SDF group (*OR* = 1.4, *p* = 0.56). When using a 1-s criterion, the number of TTS sequences fell to 65: 36 in the SDF group and 29 in the Vertical group: the odd ratio indicates a 3.8 times higher chance to observe a two-turns sequence rather than a one-turn sequence in the SDF intervention group than in the Vertical control group, but it does not reach significance (*OR* = 3.8, *p* = 0.059).

## Discussion

The present study brings pioneer data on the very first skin to skin between mother and her very premature baby. Although novel, analyses conducted in the paper are pilot, the first of their kind.

### First meeting: Not necessarily vocal

Our study focused on the very first vocal exchanges, therefore we selected dyads in which both the mother and the baby vocalized (23 out of 34 dyads). It is important to note that during the first 5 min of the first meeting after giving birth, some mothers may have not wished to speak. This very first skin-to-skin is also the very first opportunity for mothers to hold the newborn, with a place for spontaneity as it is a non-medical, non-nursing, and non-guided moment. It is a moment for intimacy, for being together, and not necessarily for speaking. Indeed, during this moment, deciding to speak or not to speak to the newborn was the mother’s choice. It seems that this choice was the expression of the mother’s preferred way of communicating, the relationship being potentially expressed through other sensory modalities: tactile (Buil et al., 2017a), kinaesthetic, visual.

### Better sleep quality

Our results showed that within the first 5 min of the very first skin to skin, very preterm newborns in the “SDF” group spent less time in a drowsy state (state 3), and more in deep sleep (state 1), than those in the Vertical control group. This result suggests that the “SDF” positioning helps preterms stabilize in restorative sleep during skin to skin, rather than stay in a state of drowsiness, considered by some authors as a transitional state, costly in energy (Als, 1982; Brazelton and Nugent, 1995; Bullinger and Goubet, 1999; Foreman et al., 2008; Devouche and Buil, 2019). This stabilization of preterm newborns’ state could rely on both an improved postural support thanks to the SDF positioning that fosters the axial winding posture with support on the neck and retroverted pelvis (Vaivre-Douret et al., 2004), but also the active behavioral support of mothers installed in SDF positioning. These mothers would intuitively help their child into deep sleep, switching rapidly from state 2 to 1, or appease him/her. From the first minutes of this renewed closeness, the SDF positioning could increase mothers’ sensitivity to their child’s signals of disorganization (such as growling, frowning, wriggling, spreading their fingers, etc.,), allowing them to respond by speaking, nursing or caressing them before they reach full disorganization.

### Premature newborn vocal presence

During this moment, that occurred on average at 3.5 days of life, newborns’ vocal production did not differ significantly between the two groups, even though vocalizations were on average longer in the Vertical group (before statistical adjustment). Our vocalizations sample was not large enough to allow us to investigate the links between length of vocalizations and states of awareness. However, we believe that the lengthier vocalizations recorded in the Vertical group could be linked to the state 3 that is found in the synactive theory of development (Als, 1982) and characterized by more grunting, which are also probably longer than other kinds of vocalizations. It would be interesting to investigate this further, by characterizing the quality of this vocalization.

One aim of this study was to better characterize the vocal meeting between mother and very preterm newborn, during the first minutes of their first ever skin to skin. Our study provides pioneering data regarding the vocal presence of the very preterm newborn during the first SSC, which represents 1.4 to 1.5 vocalizations per minute for the 23 dyads in which vocalizations did occur, regardless of the positioning. The observed frequency was much higher than that recorded by Caskey et al. (2011), because data was collected at a time dedicated to the relationship, whereas Caskey and colleagues made their recording during several hours in a crib. Vocal displays were scarcer in our very preterm newborns than in healthy term newborns installed for a cuddle in the parent’s arms or close to each other [3/min in Boiteau et al., 2021 and 2.7/min in Dominguez et al., 2016], but they were present. Indeed, it is hard to know whether these vocalizations were signs of discomfort or communication, whether they were voluntary or not, and whether they were addressed or not. Their mere existence, despite infants’ immaturity, parents’ feeling of unease and the hypermedicalization of these first moments, provides an opportunity to establish communication.

### Maternal vocal envelope

Our results showed a denser vocal presence in the SDF group than in the Vertical group (20% vs. 8%), with longer vocal interventions. This longer exposure to the mother’s voice might be important according to a recent literature review highlighting its positive impact on parenting skills (Filippa et al., 2019). This result supports the hypothesis that SSC in SDF positioning enhances and supports early vocal contact. This strategy is now recognized as a new and important Infant- and Family-Centered Developmental Care (IFCDC) strategy for the benefit of preterm infant brain development (Filippa et al., 2017; Monaci et al., 2021).

This clearly denser maternal vocal envelope could be perceived as over-stimulating for a 3–4-day preterm newborn. The SDF positioning was not conceived to promote “more” (as in “too much”) but rather “better,” closer to what would be the first meeting with a healthy term newborn. Here, judging by the analysis of the newborn’s states of consciousness, it seems that these behaviors were not perceived as over-stimulations. This question could be further explored by investigating the links between maternal voice and states of consciousness, as it was done by Filippa et al. (2018) or Saliba et al. (2020).

### Temporal coordination

In our pilot data collection, we tried to investigate the vocal temporal coordination between the mother and her very preterm newborn as Dominguez et al. (2016) and Boiteau et al. (2021) did with term-newborns. However, the few vocalizations recorded only allowed a timed analysis. Although no difference was found between both positionings regarding the amount of newborn vocalizations involved in turn-taking, descriptive results suggest more complex turn-taking in the SDF intervention group. Although not significant, this result is consistent with the denser vocal presence of mothers. We hypothesized that being more able to see the face and perceiving their newborn as an available partner, mothers in the SDF group were more likely to vocalize in a timely manner and thus create the very first vocal exchange with their baby.

Furthermore, based on a 1-s criterion, 57% of the preterm newborn vocalizations were at a temporal proximity of a mother’s vocalization in the SDF intervention group against 39% in the Vertical control group. Hence, our results show that mothers in the SDF intervention group provided a more proximal vocal envelope, but far from the 95% observed by Dominguez et al. (2016) in mothers of healthy term babies. By nature, full-term infants require the intervention of competent adults, and in this sense, it is not relevant to oppose the “natural” lives of term infants with the “unnatural” lives of hospitalized infants (Gratier and Devouche, 2017). Environments are constructed around infants but more importantly, infants, from the earliest moments of their lives, participate in this construction. The present study adds to the current knowledge and understanding that preterm infants respond to their environment too. Our results plead for more reflection on how to better adapt the environment, so that in turn, it responds to the premature baby.

### Developmental perspectives

Dyads observed in the present study were followed during the first months of life and data collected at 18 days of life, i.e., 15 days after the very first SSC have already been published in a previous paper (Buil et al., 2020). At birth, preemies in both groups did not differ in their vocal production, the main results being that mothers provide a more proximal and denser vocal envelope. Two weeks later, in the SDF Intervention Group, very preterm infants vocalized three times more, mothers vocalized, gazed at their baby’s face, and smiled more than in the Vertical Control Group, and temporal proximity of mother-infant behaviors in a one-second time frame was greater in the SDF Intervention Group (Buil et al., 2020).

The present study and the previous one plead in favor of a positive impact of a more comfortable positioning, that allows visual contact for mother-preemie interaction from the very beginning. Indeed, when offered more opportunities to be connected, mothers tended to engage more in the communication. Being able to perceive key behaviors such as open eyes, mouth movements, or being able to understand a vocal manifestation because they have a better perception of the whole story, mothers are more responsive from the very beginning. Thus, because primary intersubjectivity had the possibility to develop and so had the dyadic space (Trevarthen, 1998), mother and preemie were more able to meet and communicate 2 weeks later.

SDF positioning not only influences positively infants’ states of consciousness and mothers’ availability, thus fostering communication. Indeed, what seems to matter the most is the mother’s responsiveness, her ability to perceive the availability of her very preterm newborn to interact (Ainsworth et al., 2015) and to adjust her behavior at the level of body tonus and emotion (Ajuriaguerra, 1986).

## Long-term perspective: Supporting the preemie, the mother and the father, and a timely adjusted communication during SSC

Given the persistence of difficulties in parent-infant synchrony at 3 (Feldman and Eidelman, 2007) and 6 months GA (Forcada-Guex et al., 2011) in preterm contexts, offering SSC to support communication between the preemie and his parent as soon as possible after birth is essential. Feldman et al. (2014) pointed out that any attempt to encourage mother-infant proximity could help fragile babies and their parents to develop optimal synchrony before the end of the sensitive period. Practicing SSC with SDF positioning, rather than SSC as it is widely practiced today (Vertical positioning), offsets the paucity of parent-infant communication related to preterm birth, by enhancing both partners’ production and detection of multimodal signals, as well as their temporal coordination. Our results highlight the possibility of mother—very preterm baby vocal exchanges during skin-to-skin, especially with SDF positioning, as early as 3/4 days after birth.

The literature shows that mothers present a left side bias for carrying their baby (Donnot and Vauclair, 2005; Malatesta et al., 2019), which seems particularly stable during the first 3 months of the child’s life, regardless of culture and time (Malatesta et al., 2019). These two studies noted correlations between carrying bias and maternal empathy as well as mother’s capacity to engage emotionally in a relationship (Donnot and Vauclair, 2005; Malatesta et al., 2019). In the present study, five mothers out of 17 spontaneously chose to carry their newborn on the left side of their body during the first SSC. Hence, by allowing mothers to choose on which side their wish to carry their child each time a skin-to-skin care is proposed, SSC in SDF positioning is indeed a natural precocious way to support intuitive parenting (Papoušek and Papoušek, 2002).

In their article on the eight principles to follow in NICU, Roué et al. (2017) highlighted the need for a free 24 h/24 parental access and a psychological parental support. In their conclusion they recalled the need for future researches in this direction. More recently, Filippa et al. (2021) published a systematic review on the benefits of involving the fathers of preterm infants in early interventions in neonatal intensive care units. Their conclusion was in line with Roué et al.’s recommendations by stressing the need to develop new, multimodal and interactive interventions that provide fathers with positive contact with their preterm infants. Indeed, few studies were conducted on early father-preterm communication. However, as Boiteau et al. (2021) showed, the father and the baby meet as of the birth, why not the father and the (very) premature newborn? Benefits of SDF positioning could thus be appreciated through daily practice with fathers in NICU.

SDF positioning could promote brain-to-brain synchrony during naturalistic social interactions (Kinreich et al., 2017). SSC is described as the only developmental care dedicated to dyadic interaction (Buil, 2019), but this appears all the more obvious when both partners manage to meet (even briefly), by making eye contact, smiling and vocalizing. Even though long-term benefits of SDF positioning are yet to be investigated, our results, although based on pilot data, pleads in favor of a modification of current skin-to-skin contact practices with very preterm babies.

## Limitations

This pilot study presents some limitations. Additionally, to the number of dyads included (*N* = 34) which in itself limits the range of our results, our study was conducted in one unique level 3 NICU. A random allocation of dyads in SSC positioning would have been more optimal and would have strengthened our study design, but it would have created a risk of practice contamination. The safe implementation of SDF positioning in NICU needs to be further examined by conducting a multicentric study based on randomized sampling and including a larger group of extremely preterm infants and/or unstable very preterm infants requiring endotracheal ventilation.

## Conclusion

Very little is known about what happens between the very preterm and the parent during skin-to-skin contact, despite it being the only care entirely dedicated to the parent–infant relationship in NICUs. Seeing very preterm newborns open their eyes, attempt a smile or hearing their vocalizations are rare and short-lived moments. Yet they are essential moments, because they reveal the preterm as a fully-fledged individual, who communicates, despite immaturity and uncertain vital and developmental prognoses. They are also essential when infant and parents meet for they contribute to the creation of the first bonds.

Our study showed that whatever the positioning considered, and although we only analyzed the first 5 min of SSC in a sample of 23 dyads, very preterm newborns were able to vocalize. Indeed, during SSC, i.e., in a moment dedicated to intimacy, the very preterm newborn was vocally present and provided opportunities to establish communication. Moreover, SDF positioning supported mothers’ vocal responsiveness to the vocal presence of their newborn, thus fostering the beginning of adapted and coordinated vocal responses, and feeding intersubjective intimacy.

Providing early communicational pathways for parents and very preterm infants, as early as possible, enables initial interactive experiences, providing a less atypical, less dysregulated, and more consistent environment. Our pioneer data sheds light on the way a mother and her very preterm vocally meet, and constitutes a pilot step in the exploration of innate intersubjectivity in the context of very preterm birth. Future studies are needed to explore other ways by which the very preterm connects to his/her partner, thus creating a dyadic intersubjective space, i.e., vision, tonic postural dialogue, rhythm, touch, and olfaction.

## Data availability statement

The datasets presented in this article are not readily available because it contains sociodemographic and medical information that cannot be shared consistently with GDPR requirements. Requests to access the datasets should be directed to devouche7@gmail.com.

## Ethics statement

All mothers were offered to participate to the research study on a voluntary basis, within the first two days postpartum, and in all cases before the first SSC session. Every mother was informed of the research by a letter in the NICU. Mothers gave a written informed consent before participating. Written informed consent was obtained from the mothers for the publication of any potentially identifiable images or data included in this article. An initial information-based meeting was organised prior to data collection. This research was approved by the French Local Ethics Consulting Committee for the Protection of Persons (IRB n°2015120001072).

## Author contributions

AB and ED contributed to the conception and design of the study and wrote the first draft of the manuscript. AB collected the data. ED performed the statistical analysis. All authors contributed to the article and approved the submitted version.

## Funding

This work was supported by four research grants by Institut Universitaire de France, Fondation Mustela, Fondation pour la Recherche en Psychomotricité et Maladies de Civilisation, and Fondation des Treilles. Baby wraps were provided by L’école à porter—JPMBB.

## Conflict of interest

The authors declare that the research was conducted in the absence of any commercial or financial relationships that could be construed as a potential conflict of interest.

## Publisher’s note

All claims expressed in this article are solely those of the authors and do not necessarily represent those of their affiliated organizations, or those of the publisher, the editors and the reviewers. Any product that may be evaluated in this article, or claim that may be made by its manufacturer, is not guaranteed or endorsed by the publisher.

## Footnotes

^1^ http://www.noldus.com

^2^ https://audacity.sourceforge.net/

## Abbreviations

APIB: Assessment of preterm infants’ behavior scale
IFCDC: Infant- and Family-Centered Developmental Care
GA: Gestational age
NICU: Neonatal intensive care unit
SDF: Supported diagonal flexion
SSC: Skin-to-skin contact
